# Bowel perforation secondary to illegally induced abortion: a tertiary hospital experience in Tanzania

**DOI:** 10.1186/1749-7922-7-29

**Published:** 2012-09-01

**Authors:** Joseph B Mabula, Phillipo L Chalya, Mabula D Mchembe, Albert Kihunrwa, Anthony Massinde, Alphonce B Chandika, Japhet M Gilyoma

**Affiliations:** 1 Department of Surgery, Catholic University of Health and Allied Sciences, Bugando, Mwanza, Tanzania; 2 Department of Surgery, Muhimbili University of Health and Allied Sciences, Dar Es Salaam, Tanzania; 3 Department of Obstetric & Gynaecology, Catholic University of Health and Allied Sciences, Bugando, Mwanza, Tanzania

**Keywords:** Bowel perforation, Illegally induced abortion, Surgical management, Tanzania

## Abstract

**Background:**

Bowel perforation though rarely reported is a serious complication of induced abortion, which is often performed illegally by persons without any medical training in developing countries. A sudden increase in the number of patients in our centre in recent years prompted the authors to analyze this problem. The study was conducted to describe our own experiences in the surgical management of these patients.

**Methods:**

This was a retrospective study involving patients who were jointly managed by the surgical and gynecological teams at Bugando Medical Centre (BMC) for bowel perforation secondary to illegally induced abortion from January 2002 to December 2011. The statistical analysis was performed using SPSS version 17.0.

**Results:**

A total of 68 patients (representing 4.2% of cases) were enrolled in the study. Their ages ranged from 14 to 45 years with a median age of 21 years. Majority of patients were, secondary school students/leavers (70.6%), unmarried (88.2%), nulliparous (80.9%), unemployed (82.4%) and most of them were dependent member of the family. Previous history of contraceptive use was reported in only 14.7% of cases. The majority of patients (79.4%) had procured the abortion in the 2^nd^ trimester. Dilatation and curettage (82.4%) was the most common reported method used in procuring abortion. The interval from termination of pregnancy to presentation in hospital ranged from 1 to 14 days (median 6 days ). The ileum (51.5%) and sigmoid colon (22.1%) was the most common portions of the bowel affected. Resection and anastomosis with uterine repair was the most common (86.8%) surgical procedure performed. Complication and mortality rates were 47.1% and 10.3% respectively. According to multivariate logistic regression analysis, gestational age at termination of pregnancy, delayed presentation, delayed surgical treatment and presence of complications were significantly associated with mortality (P<0.001). The overall median length of hospital stay (LOS) was 18 days (1day to 128 days ). Patients who developed complications stayed longer in the hospital, and this was statistically significant (P=0.012).

**Conclusion:**

Bowel perforation following illegally induced abortion is still rampant in our environment and constitutes significantly to high maternal morbidity and mortality. Early recognition of the diagnosis, aggressive resuscitation and early institution of surgical management is of paramount importance if morbidity and mortality associated with bowel perforation are to be avoided.

## Background

Globally, illegally induced abortion constitutes a major public health problem and in Africa particularly, the picture is of increasingly hospital admissions for abortion complications and a distressingly high rate of maternal morbidity and mortality due to abortions [1,2]. Worldwide, there are 30-50 million induced abortions that result in the death of 80,000 - 110,000 women of which an estimated 34,000 are in Sub–Saharan Africa [1,3]. In settings where access to abortion is highly restricted and desire to regulate fertility is low, deaths due to abortion is a major contributor to maternal mortality [3]. In Tanzania, the law on abortion is highly restrictive and does not permit termination of pregnancy except when it is needed to save the life of a woman [4]. Consequently, women frequently resort to clandestine abortion performed by unskilled practitioners, leading to high rates of maternal mortality and morbidity. The most common reasons for induced abortion are unwanted pregnancy, having lactating small child, health problems, economic and social or family problems that forced women to induce abortion [5-7]. More proximate causes include poor access to contraceptives and contraceptive failure. Family planning programs are very important for prevention of unwanted pregnancy. Lack of education, social stigma and other barriers to abortion, force women to seek abortion in secrecy at a high cost, leaving the poorest, least educated women to unskilled and highly unscrupulous executors and hence the greatest risk of injury [8].

Complications resulting from unsafe induced abortion are a major cause of maternal mortality, morbidity, prolonged hospitalization and reproductive failure in developing countries including Tanzania [4]. The most common complications of induced abortion include genital sepsis, haemorrhage, pelvic infection with peritonitis and abscess formation, uterine and bowel perforations [9,10].

Bowel perforation is a rare but serious complication of induced abortion, which is often performed illegally by persons without any medical training in developing countries [11]. The incidence of bowel injury has varied between 5 to 18% cases in different studies [12-14]. The high incidence of perforation in most developing countries has been attributed to late diagnosis resulting from late presentation to health facilities [15].

The bowel may be injured with the curette, ovum forceps or uterine sound, or even the plastic canula. Bowel perforation occurs when the posterior vaginal wall is violated, allowing the instrument to pierce the underlying structures [16]. The ileum and sigmoid colon are the most commonly injured portions of the bowel due to their anatomic location [9,16-20].

The management of cases with intestinal injuries following induced abortion poses some major challenges to general surgeons and gynecologists practicing in resource-limited countries [9]. Surgery is considered the treatment of choice in order to improve the chances of survival of patients with this condition. However, late presentation and diagnosis coupled with lack of diagnostic facilities, inadequate preoperative resuscitation and delayed operation are among the hallmarks of the disease in most developing countries including Tanzania [9,18]. Early recognition and prompt surgical treatment of bowel perforation following illegally induced abortion is of paramount importance if morbidity and mortality associated with bowel perforation are to be avoided [9]. A successful outcome is obtained by prompt recognition of the diagnosis, aggressive resuscitation and early institution of surgical management.

Despite the documented increasing safety of the procedure, many women have limited access to abortion services due to logistic and social obstacles [21]. Hence, complications related to illegally induced abortion such as bowel perforations are believed to still be rampant in our environment. A sudden increase in the number of admissions of patients with bowel perforation following illegally induced abortions in our setting prompted the authors to analyze this problem. The aim of this study was to describe the pattern and treatment outcome of bowel perforations following illegally induced abortions in our local setting.

## Methods

This was a retrospective study involving patients who were jointly managed by the surgical and gynecological teams at Bugando Medical Centre (BMC) for bowel perforation secondary to illegally induced abortion from January 2002 to December 2011. BMC is a tertiary and teaching hospital for the Catholic University of Health and Allied Sciences-Bugando (CUHAS-Bugando). It is located in Mwanza city and has a bed capacity of 1000. The study included all patients who were managed by the surgical and gynecological teams at our centre for bowel perforation secondary to illegally induced abortion during the study period. Patients with incomplete data were excluded from the study. Information on socio-demographic data, parity, gestational age at termination of pregnancy, interval from termination of pregnancy to presentation in hospital, clinical presentation, perforation-surgery interval, site of intestinal injury, management and clinical outcome was obtained from medical record database and from patients’ files, theatre and surgical and gynecological ward registries. All patients were first seen by the gynecologists at the Accident and Emergency department who made the diagnosis based on clinical findings. Radiological, haematological and biochemical investigations were carried out after initial fluid resuscitation. The patients were optimized clinically and commenced on broad spectrum antibiotics active against anaerobes, gram positive and gram negative organisms. The surgical team was then invited to join in the management. Exploratory laparotomy was carried out with repair of uterine and intestinal injury as deemed appropriate by the operating surgeon. Both teams were usually involved in the postoperative management and outpatient follow-up.

### Statistical analysis

The statistical analysis was performed using statistical package for social sciences (SPSS) version 17.0 for Windows (SPSS, Chicago IL, U.S.A). The median and ranges were calculated for continuous variables whereas proportions and frequency tables were used to summarize categorical variables. Continuous variables were categorized. Chi-square (Χ^2^) test were used to test for the significance of association between the independent (predictor) and dependent (outcome) variables in the categorical variables. The level of significance was considered as P<0.05. Multivariate logistic regression analysis was used to determine predictor variables that predict the outcome.

### Ethical consideration

Ethical approval to conduct the study was obtained from the CUHAS-Bugando/BMC joint institutional ethic review committee before the commencement of the study

## Results

Out of 1619 patients who presented with induced abortion-related complications during the study period, 79 patients underwent exploratory laparotomy due to associated bowel perforation. Of these, 11 patients were excluded from the study due to incomplete data. Thus, a total of 68 patients representing 4.2% of cases were enrolled in the study. Their ages ranged from 14 to 45 years with a median age of 21 years. The modal age group was 21-25 years accounting for 47.1% of cases**.** Most patients (61.8%) came from urban areas in Mwanza city and other regions in northwestern Tanzania. Majority of patients were, secondary school students/leavers (70.6%), unmarried (88.2%), nulliparous (80.9%), unemployed (82.4%) and most of them were dependent member of the family.

The gestational ages of pregnancies at induced abortion admitted to by the patients ranged between 5 to 24weeks. The median gestational age at termination of pregnancy was 13weeks. Previous history of contraceptive use was reported in only 14.7% of cases. The majority of patients (79.4%) had procured the abortion in the 2^nd^ trimester while 14 (20.6%) patients had theirs in the 1^st^ trimester. Analysis of the results showed that the majority of patients (77.9%) had no previous history of pregnancy terminations (Table 1). Dilatation and curettage was the most common method used in procuring abortion in 56 (82.4%) patients. Methods used in procuring abortion were not documented in 12 (17.6%) patients.

**Table 1:** Distribution of patients according to patient’s characteristics

**Variable**	**Response**	**Number of patients**	**Percentage**
**Age**	< 15	2	2.9
	16-30	56	82.4
	>30	10	14.7
**Area of residence**	Urban	42	61.8
	Rural	26	38.2
**Parity**	Nulliparous	55	80.9
	1-3	10	14.7
	>3	3	4.4
**Marital status**	Unmarried	60	88.2
	Married	8	11.8
**Education status**	No formal education	6	8.8
	Primary	9	13.2
	Secondary	48	70.6
	Tertiary	5	7.4
**Occupation**	Employed	12	17.6
	Unemployed	56	82.4
**Previous history of contraceptive use**	Yes	10	14.7
	No	58	85.3
**Previous history of induced abortion**	No	53	77.9
	1	6	8.8
	≥2	5	7.4
	Not documented	4	5.9
**Gestational age**	1^st^ Trimester	14	20.6
	2^nd^ Trimester	54	79.4

The majority of abortion providers, 56 (82.3%) reported was health care workers described as medical doctors by patients. Reasons for procuring abortion are shown in Table 2 below. The place where abortions were conducted was known in only 23 (33.8%) patients and this included private health facilities in the majority of patients, 20 (86.9%). The place was not documented in 45 (66.2%) patients.

**Table 2:** Distribution of patients according to reasons for termination of pregnancy

**Reason for termination of pregnancy**	**Frequency**	**Percentage**
Fear of expulsion from school	62	91.2
Does not want patents or others to know about the pregnancy	60	88.2
Too young to have a child	45	66.1
Has relationship problem	34	50.0
Cannot afford a child	23	33.8
Reasons not documented	18	26.5

The duration of illness ranged from 1 to 14 days with a median duration of 6 days . Twenty (29.4%) patients presented within twenty-four hours of onset of symptoms (early presentation) and 44 (64.7%) patients presented after 24 h (late presentation). The duration illness was not documented in 4 (5.9%) patients. Distribution of patients according to clinical presentation is shown in Table 3.

**Table 3:** Distribution of patients according to clinical presentation

**Clinical presentations**	**Frequency**	**Percentage**
Abdominal pain	68	100
Fever	42	61.8
Vaginal bleeding	31	45.6
Offensive vaginal discharge	28	41.2
Abdominal distention	23	33.8
Diarrhea	18	26.5
Vomiting	12	17.6
Passing feces through vagina	9	13.2
Visible loops of bowel through vagina	8	11.8
Signs of peritonitis	68	100

The median haemoglobin level and white blood cell count on admission were 10.8 g/dl (range 6.8-13.9 g/dl) and 11.5 x 10^9^ cells/l (range 3.6- 34.2 x 10^9^ cells/l) respectively. The haemoglobin level was less than 10 g/dl in 38 (55.9%) patients. Serum electrolytes revealed hypokalaemia and hyponatraemia in 23 (33.8%) and 18 (26.5%) patients respectively. Serum electrolytes result was not documented in 15 (22.1%) patients. Thirty-two of 68 (47.1%) patients in whom plain abdominal x-rays were taken had pneumoperitoneum. Abdominal ultrasound done in 63 (92.6%) patients detected free peritoneal collections in 49 (77.8%) patients.

The perforation-surgery interval was within 24 h in 16 (23.5%) patients and more than 24 h in 52(76.5%) patients. The interval between presentations at the Accident and Emergency department and surgery (waiting time) ranged from 18 h with a median of 4 h.

All patients in this study underwent exploratory laparotomy. At laparotomy adhesion-exudative and fibrinous, were present between the pelvic organs, the bowels and the anterior abdominal wall. Abscess in the adnexa were in association with tubo-ovarian complexes. The abdominal cavity was heavily contaminated (generalized peritonitis) in 48 (70.6%) patients while in 20 (29.4%) patients the peritoneal cavity was having minimal contamination (localized peritonitis). The amount of pus/faecal matter drained from the peritoneal cavity reflected the extent of peritoneal contamination and ranged from 150 to 2500 mls with a mean of 725 ± 231 mls. It was less than 1000 ml in 21 (30.9%) patients and more than 1000 mls in 47 (69.1%) patients. Associated haemoperitoneum was reported in 8 (11.8%) patients and the amount ranged from 100 to 1500 mls (mean 456± 673 mls). The ileum was involved in 35 (51.5%) patients and jejunum in 14 (20.6%) patients. Fifteen (22.1%) patients had injury to the sigmoid colon and 4 (5.9%) to the recto-sigmoid. The affected bowel was viable in 51 (75.0%), gangrenous in 18 (26.5%) and prolapsed through the vagina or uterine perforations in 10 (14.7%) patients. Associated uterine injuries was noted in all patients and ranged from perforations to outright lacerations positioned posteriorly 39 (57.4%), lateral 16 (23.5%), fundal 10 (14.7%) and anteriorly 3 (4.4%).

Bowel re-section and end to end anastomosis was the most common surgical procedure performed accounting for 86.8% of cases. Surgical procedures performed among patients with bowel perforation following illegally induced abortion are depicted in Table 4**.** The duration of operation was documented in 62 (91.2%) patients and ranged from 70 to 120 min with a median duration of 82 min. The duration of operation was not known in six (8.8%) patients.

**Table 4:** Distribution of patients according to surgical procedures performed

**Surgical procedures performed**	**Frequency**	**Percentage**
Bowel resection and end to end anastomosis	59	86.8
Uterine perforation repair	53	77.9
Repair of bowel perforations	12	17.6
Hysterectomy	8	11.8
Adnexectomy	7	10.3
Bowel perforation repair/bowel resection + colostomy	5	7.4

A total of 72 postoperative complications were recorded in 32 patients giving a complication rate of 47.1%. Surgical site infection was the most common postoperative complication accounting for 38.9% of cases (Table 5).

**Table 5:** Distribution of patients according to postoperative complications (N=72)

**Postoperative complications**	**Frequency**	**Percentage**
Surgical site infections	28	38.9
Postoperative pyrexia	14	19.4
Postoperative diarrhea	8	11.1
Wound dehiscence	5	6.9
Enterocutaneous fistula	4	5.6
Peritonitis	4	5.6
Septic shock	4	5.6
Pelvic abscess	3	4.1
Paralytic ileus	2	2.8
Total	72	100

In this study, seven patients died giving a mortality rate of 10.3%. According to multivariate logistic regression analysis, gestational age at termination of pregnancy, delayed presentation, timing of surgical treatment (delayed surgical treatment)and presence of postoperative complications were significantly associated with mortality (P<0.001).

The overall length of hospital stay (LOS) ranged from 1 day to 128 days with a median of 18 days . The LOS for non-survivors ranged from 1 to 10 days (median = 4 days ). The length of ICU stay ranged from 1 to 21 days (median = 8 days ). According to multivariate logistic regression analysis, patients who developed complications stayed longer in the hospital, and this was statistically significant (P=0.012).

Of the survivors (61), fifty-six (82.4%) patients were discharged well, four (6.6%) patients were discharged against medical advice (DAMA) and the remaining one (1.6%) patient was discharged with permanent colostomy due to severe injury to the recto-sigmoid portion of the colon. Out of 61 survivors, 26 (42.6%) patients were available for follow up at 3months after discharge and the remaining 35 (57.4%) patients were lost to follow up.

## Discussion

Bowel perforation secondary to illegally induced abortion though rare and uncommon in developed world is a significant and major cause of maternal morbidity and mortality in countries like Tanzania where abortion laws are still restrictive and most abortions are performed clandestinely and illegally by unqualified personnel [3,15]. The incidence of abortion-related complications such as bowel injuries has been reported in most developing countries to be increasing at an alarming rate [22]. The rate of bowel perforation as a complication of induced abortion has been reported in literature to range from 5% to 18% of all abortion-related complications [12-14]. However, as in other iatrogenic surgical problems, many cases may have been unreported because of its medico-legal implications [9,23]. In this study, the rate of 4.2% of bowel perforations may actually be an underestimate and the magnitude of the problem may not be apparent because many cases are not reported for fear of been arrested by police. Several other cases may also have been treated in private hospitals which were not included in the present study. Exclusion of large number of patients in this study as a result of lack of enough data may have also contributed to the underestimation of the magnitude of the problem.

In keeping with other studies [2,9,24,25], majority of our patients who underwent induced abortion were young, secondary school students/leavers, unmarried, nulliparous, unemployed and most of them were dependent member of the family. This finding is contrary to what was observed by Rehman et al. [26] who reported that most of the women were married and had five or more children. The majority of patients in the present study presented themselves for abortion when the pregnancy was advanced and, therefore requiring relatively more complicated termination procedure which only a specialist may handle. But because of socio-economic, cultural and law restrictive reasons most of these women fear of revealing their pregnancy and as a result fall prey to unqualified and inexperienced people who perform such illegal procedures under substandard unhygienic places.

The majority of patients in this study came from urban areas, which is in agreement with other studies done elsewhere [3-5,9,11,15-17]. Previous studies have shown that premarital sexual intercourse is practiced much in urban than in rural areas probably because of increasing urbanization that broke down cultural barriers and predisposed to increased sexuality [27]. This needs to be studied further so that effective intervention strategies for positive behavioral change will be mounted.

In this study, the rate of contraceptive use was as low as 14.7% which is comparable with other studies done in developing countries [4,24,28-30]. Low contraceptive uptake may be due to fear about the safety of contraceptives, lack of knowledge about family planning, religious believes and lack of access to services. This calls for proper training and continuing education for awareness on abortion and its complications.

In the present study, more than 70% of patients had procured the abortion in the 2^nd^ trimester which is consistent with other studies [29,30], but at variant with Enabudoso et al. [31] in which women sought abortion in the first trimester. Ignorance and inability to take quick decision regarding termination of an unwanted pregnancy compel a large number of women to seek illegally induced abortion in the second trimester from unauthorized person in unrecognized places. The delay in procuring abortion in the present study may be due to the restrictive abortion laws in this country, the secrecy associated with abortion and the religious and social norms that do not accommodate abortion practice. Also, lack of financial support may have contributed to delay in procuring abortion.

Women's reasons for seeking abortion were discussed in several studies [9,24,29-31]. These included inappropriate timing of the pregnancy, fear of expulsion from school, financial difficulties, and uncertainties about the partner. In this study, fear of expulsion from school was the most common reason for terminating pregnancy.

As reported by many authors [15,17,30,31], majority of patients in the present study presented late in poor general condition. This was found to be the most important factor influencing the outcome of surgical procedure as also emphasized by a number of authors [9,15,30]. In resource-poor countries, difficulties in diagnosis, lack of awareness of the disease and delayed referral to tertiary hospital often result in delayed presentation to a hospital

Surgical intervention is considered to be the gold standard treatment for patients with bowel perforation following induced abortion [9]. In this study, all patients underwent surgical treatment which is in keeping with other studies [9,11,16-20,26,32,33]. One of the many factors affecting the surgical outcome in patients with bowel perforation is time interval from perforation to laparotomy [9,15]. Early surgery can minimize the complications while delayed surgery leads to severe peritonitis and septic shock. In the present study, the majority of patients were operated more than 24 h after the onset of illness. Similar observation was reported by other studies done in developing countries [4,9,30]. Delayed definitive surgery in the present study may be attributed to late presentation due to lack of accessibility to health care facilities, lack of awareness of the disease as a result some patients with bowel perforation following induced abortion may decide to take medications in the pre-hospital period with hope that the symptoms will abate. It is also possible that some clinicians managing the patients initially may not have considered perforation as a possible diagnosis leading to delayed referral to tertiary care hospital.

In keeping with other studies [9,16-20], the ileum and the sigmoid colon were the most common parts of the bowel affected. The relative fixity of these portions of the bowel has been suggested as a possible reason for this.

Early surgical interference is the optimal treatment option for perforation. However, the type of surgery to be applied is controversial [9]. The surgical management of small intestinal injuries is fairly straightforward with minimal sequalae. Our practice in managing these patients is a simple closure in solitary perforations and segmental intestinal resection and primary anastomosis in multiple perforations or gangrenous bowel. The management of large bowel injury is more controversial [9,18]. This is more so when the left colon is involved. A simple colostomy has been reported to be the safest approach in the management of these injuries. Other options include primary repair, resection and primary anastomosis, and repair with a proximal protective colostomy. A simple colostomy is easier and faster to accomplish in these poor surgical risk patients. However, the major drawback of colostomy is the need for a second operation to restore intestinal continuity, the specialized care before closure and the attendant cost which reduces its popularity [34,35]. The challenge is even more conspicuous in a developing country like Tanzania where resources for caring of patients with colostomy are limited. The management of stoma remains difficult in developing countries because of the shortage of suitable equipment in this respect and peristomal ulceration remains a major problem [35]. Experiences in our centre are primary repair and resection and primary anastomosis in case of viable bowel, whereas colostomy is reserved after resection of a gangrenous large bowel.

The overall complications rate in this series was 47.1% which is higher compared to what was reported by Thapa et al. [36]. High complications rate was also reported by Saleem & Fikree [37] in Pakistan. This difference in complication rates can be explained by differences in antibiotic coverage, meticulous preoperative care and proper resuscitation of the patients before operation, improved anesthesia and somewhat better hospital environment. As reported by Rehman et al. [26], surgical site infection was the most common postoperative complication in our study. High rate of surgical site infection in the present study may be attributed to contamination of the laparotomy wound during the surgical procedure.

In this study, mortality rate was 10.3% which is higher than that reported by Bhutta et al. [38]. High mortality rate in this study is attributed to high gestational age at termination of pregnancy, late presentation, delayed surgical treatment and postoperative complications.

The overall median length of hospital stay was 18 days , a figure which is lower than that reported by Rehman et al. [26]. Our overall median length of hospital stay was significantly long in patients who developed complications postoperatively. Prolonged length of hospitalization results in consumption of large amounts of healthcare resources such as personnel, theatre space, medications, and hospital beds.

Self-discharge against medical advice is a recognized problem in our setting and this is rampant, especially amongst patients with complications of illegally induced abortions [39]. Similarly, poor follow up visits after discharge from hospitals remain a cause for concern. These issues are often the results of poverty, long distance from the hospitals and ignorance.

## Conclusion

Bowel perforation following illegally induced abortion is still rampant in our environment and constitutes significantly to high maternal morbidity and mortality. The majority of patients in this study were young, secondary school students/leavers, unmarried, nulliparous, unemployed and most of them presented late to our centre in poor general condition. Early recognition of the diagnosis, aggressive resuscitation and early institution of surgical management is of paramount importance if morbidity and mortality associated with bowel perforation are to be avoided. Appropriate measures focusing at reducing the occurrence of illegally induced abortion are vital in order to reduce the incidence of bowel perforation following illegally induced abortion in this region.

## Competing interests

The authors declare that they have no competing interests.

## Authors contributions

JBM conceived the study and participated in the literature search, writing of the manuscript and editing the article. PLC, MDM, GG, AK, AM, ABC, participated in Study design, data analysis, manuscript writing & editing. In addition PLC submitted the manuscript. JMG was involved in study design, data analysis, coordination and supervision of manuscript writing & editing. All the authors read and approved the final manuscript.
